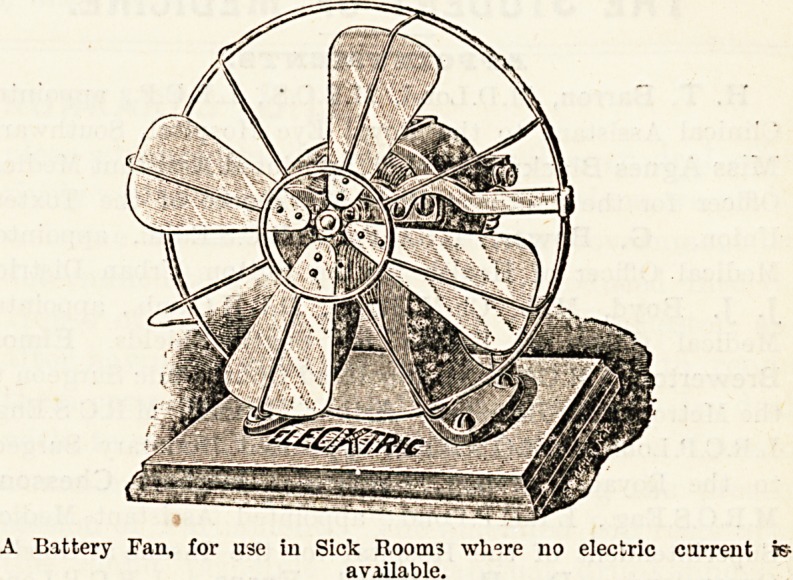# Practical Departments

**Published:** 1901-05-18

**Authors:** 


### PRACTICAL DEPARTMENTS.

### ELECTRIC FAN MOTORS.

(The Improved Electric Glow Lamp Co. Ltd., 103 Queen Victoria Street, London, E.C.)

Where the electric light is already installed the electric fan motor offers a simple method of keeping the air in circulation, while by means of the ventilating fan, a constant supply of fresh air from outside may be secured. These fans are found of immense value on board the transports and hospital ships which are bringing home wounded and sick from South Africa, especially in the tropics, where the difficulty of regulating the temperature to that suitable for fever cases is extreme. One of the firms supplying such fans is the Improved Electric Glow Lamp Company, Limited, and their catalogue offers a large variety of direct and alternating current desk, ceiling, and ventilating fans. A strongly built fan, designed for hard use, and requiring little attention, has a diameter of 12 or 16 inches; voltage, 110 or 220; amperage, ·25, ·33, ·42 or ·65. The speed varies from 1,350 to 1,650. There is also a midget direct-current ceiling fan,

A Battery Fan, for use in Sick Rooms where no electric current is available.

with blades of 24 inches sweep, and 500 revolutions in the minute; it can be used with a wall-controlling switch. Some of the ceiling fans are very highly finished on the model of chandeliers. When no electric current is available, a small battery fan will be found an immense boon in the sick room; the one in the illustration runs 150 hours without requiring to be re-charged. The diameter is six or ten inches, and it can of course be placed anywhere in the room. One of the large buildings supplied by this firm is the Carlton Hotel, where we understand the fans work very satisfactorily.

## Figures and Tables

**Figure f1:**